# Effect of task specific circuit training on Gait parameters and mobility in stroke survivors

**DOI:** 10.12669/pjms.345.15006

**Published:** 2018

**Authors:** Qurat-ul-Ain, Arshad Nawaz Malik, Umama Haq, Sehrish Ali

**Affiliations:** 1 Dr. Qurat-ul-Ain, MSNMPT, Department of Physical Therapy, Pakistan Railway Hospital, Riphah International University, Islamabad, Pakistan; 2 Dr. Arshad Nawaz Malik, PhD, Department of Physical Therapy, Pakistan Railway Hospital, Riphah International University, Islamabad, Pakistan; 3 Dr. Umama Haq, MSNMPT, Department of Physical Therapy, Pakistan Railway Hospital, Riphah International University, Islamabad, Pakistan; 4 Dr. Sehrish Ali, MSOMPT, Department of Physical Therapy, Pakistan Railway Hospital, Riphah International University, Islamabad, Pakistan

**Keywords:** Circuit gait training, Gait Parameters, Timed get up and go, Stroke

## Abstract

Objective of the study was to investigate effects of task specific circuit gait training to improve gait parameters and mobility among sub-acute and chronic stroke patients. A randomized control trial was conducted on stroke survivors of either gender being capable of standing 10 seconds and having 2-4 score on Rankin Modified Scale. Sample comprised of 30 participants randomly assigned into two groups. Training was given for a session of 40-50 minutes for 3-4 days/week for six weeks. Timed get up and go test (TUG), Cadence, Step Length and Step Width assessed measures of concern. The sample included 16 males and 14 females with mean age of 54.10 ± 10.10 years. After six weeks, significant improvement was recorded in TUG (*p*=0.014). Cadence (*p*=0.001), step length (*p*<0.001) and step width (*p*=0.009) were also significantly improved. Circuit gait training improves mobility and gait in stroke patients.

## INTRODUCTION

Worldwide number of stroke patients is rising and becoming leading cause of long term disability.^1^ Stroke survivors require care over long periods of time to deal with life time disabilities resulting from stroke.^2^ Out of 50 million stroke patients worldwide 25-74% relies on assistance or is completely dependent on caretakers for basic activities of daily life.^3^ Pettersen R et al. found that after rehabilitation around 62% of stroke survivors are still not independent in activities of daily life and even after three years post stroke 32 % could not achieve instrumental activities of daily living.^4^ Independence in activities of daily life is highly impacted by walking, one of the initially lost skill by almost 80 % of stroke survivors.^5^

Mobility problems in stroke patients is very common and self-reported issues related to mobility are most common long standing need of stroke survivors.^6^ An estimated one fifth stroke survivors having chronic stroke have substantial deterioration in mobility^7^ & not as much as 50% could manage walking independently in community.^8^

A meta-analysis (12 RCT, N=501) reported progressive and task oriented training to be more effective to improve walking distance & gait speed as compared to usual individual rehab care.^9^ Unfortunately, Neuro physiotherapists are hindered in the execution of rigorous, task-specific exercises by lack of time due to inadequate & ineffective usage of human resources.^10^ Wevers L et al. in their meta-analysis concluded that task-oriented circuit training (CCT), having series of workstations forming a circuit, showed better outcomes in terms of walking distance, speed of gait and the time up and go test, compared to conventional therapy.^11^ Current study was planned to investigate effects of task specific circuit training to improve gait parameters and mobility.

## METHODS

The effect of task specific circuit gait training was investigated by single blinded randomized control trial. Patients falling on set criterion were randomly allocated into two groups, control and interventional groups. Conventional standard rehab protocol was given to the control group while circuit gait training was provided to the experimental group. Blind assessor measured the baseline data and then after every two weeks of training. Total duration of training was six weeks.

Study was conducted from 01^st^ Jan, 2016 to 31^st^ Jul, 2016. Study was approved by ethical committee of Riphah College of Rehabilitation Sciences. Sample of the study was comprised of 36 stroke patients and met the sample size used in previous studies. Out of 36 patients 30 patients could successfully complete six weeks of training and six patients were dropped out due to different reasons. Sample was equally distributed among the two groups. Stroke survivors of both gender, age between 30-70 years, any type of stroke, meeting criterion score for Modified Rankin Scale (MRS Score: 2-4) and able to perform 10 second independent standing were included in the sample after signing informed consent. Patients having cognitive/communication problems, severe abnormal synergies, contractures, trauma and fractures were excluded during initial evaluation.

## RESULTS

### Treatment protocol

Traditional Gait Training Group (TGTG): Traditional gait training exercises were given to the control group for four days a week with session duration 40-50 minutes. This treatment was continued for a period of six weeks.

### Circuit Gait Training Group (CGTG)

Eight work stations of different activities related to balance and gait were defined at each work stations. These activities included tandem walk, one leg standing, one leg standing on foam, walking on different surfaces, stair climbing, standing on balance board, walking on a set pattern on floor and moving through obstacles. Patient practiced each task on station for 4-5 minutes. Total time for the session was 40-50 minutes and continued four days a week over a period of six weeks. All work stations were supervised by therapist.

**Fig. 1:**
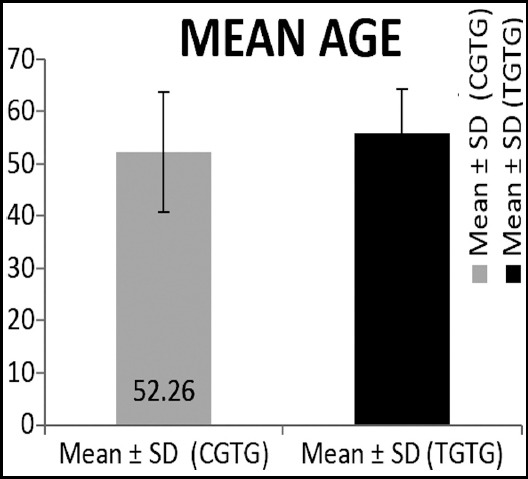
Comparison of mean of the participants among both control and experimental groups.

Out of total participants 33.3% had hemorrhagic stroke and 66.7 percent had ischemic stroke. Sample had 53.3 % male patients and 46.7 % females. Mean age of the participants was 54.10 ± 10.10 whereas mean age of the participants among the two groups; circuit gait training group and traditional gait training group is shown in Fig.1. Sample had 50% of the participants having right hemiplegia and equal amount of participants with left hemiplegia; with equal distribution among the two training groups.

After six weeks of intervention significant improvement was seen in TUG and Gait parameters (cadence, step length and step width) scores. Data was collected using valid standardized assessment tool for static and dynamic mobility; Timed Get Up and Go Test. The measurements were taken at baseline, after two, four and six weeks for TUG whereas Gait Parameters were assessed only pre and post intervention.

### Statistical analysis

Statistical analysis was done using SPSS 21. Normality tests were applied at baseline and showed homogenous data distribution so the test used for inferential analysis was Independent sample t-test for gait parameters and ANOVA for TUG to compare results across the groups (Table I and II).

**Table-I:** ANOVA used to compare timed get up and go test across both groups.

Variable	CGTG (Mean ± SD)	TGTG (Mean ± SD)	P-Value
TUG Baseline	26.01 ± 8.5	29.51 ± 9.5	0.928
TUG after 2 weeks	23.43 ± 8.5	28.98 ± 10.4	0.123
TUG after 4 weeks	20.87 ± 8.02	28.41 ± 11.9	0.052
TUG after 6 weeks	16.57 ± 7.1	26.22 ± 12.3	0.014*

**Table-II:** Independent-t test used to compare cadence, step length and step width across both groups.

Variable	CGTG (Mean ± SD)	TGTG (Mean ± SD)	P-Value
Cadence Baseline	71 ± 8.9	68.73 ± 8.3	0.359
Cadence (6 weeks)	87.86 ± 11.9	74.06 ± 7.5	0.001**
Step Width Baseline	22.26 ± 4.9	20.21 ± 3.1	0.191
Step Width Baseline (6 weeks)	14.50 ± 4.6	18.70 ± 2.7	0.009**
Step Length Baseline	14.78 ± 3.9	12.29 ± 2.7	0.053
Step Length Baseline (6 weeks)	21.23 ± 3.9	14.11 ± 2.9	<0.001***

## DISCUSSION

Current study recommends task specific circuit training to be more effective as compared to conventional therapy in improving gait and mobility. Ingrid G.L. van de Port and colleagues in their systematic review (21 high quality RCT’s) also reported a significant effect of task oriented gait training on gait parameters.^9^

Results of the study show that there is an improvement in the gait parameters after 6-week training but this improvement is more marked and significant in the interventional group (circuit gait training group) in comparison with control group (traditional gait training). A prominent increase in step length also shows that balance is also improving with gait training. More over as the patient gains balance and improves step length he/she moves towards a more stable gait with reduced or near to normal base of support thus reducing the step width. With increase in step length and reduction in step width there was a prominent gain in cadence as well. Another study published in 2009 by Ingrid G.L et al concludes “task-orientated CCT holds great potential for the rehabilitation of people after stroke, allowing the training schedule to be customized to the individual status of each participant.^2^”

A Systematic review by Wevers et al. used five studies (12-17) (n=244) to analyze response of task specific circuit training on time up and go test. Time up and go test was performed by the standard method of the test described by “Podsiadlo and Richardson”. A significant homogeneous SES was found in favor of circuit class training when compared with control group *P*=0.047. Study Dean et al.’s^12^, also reported that circuit designed for task oriented training shows better results and prominent decrease in time for time up and go test.

A similar study by Kim also showed that in comparison of individual/ task specific gait training, circuit training was effective in improving gait and balance. A study by Bonggil Kim B et al. shows that circuit training in a group shows better result in balance control of stroke patients than in individual training.^13^

As stroke patients have to deal with lifelong disabilities and quite often stroke patient’s individual status is not stable over time, it is essential to look into ways that could possibly lead to a transformation in chronic stroke care.

## CONCLUSION

Task specific circuit gait training embraces pronounced potential for gait rehabilitation of stroke survivors. Practicing specific task related to gait and balance in a circuit manner improves both mobility and gait by improving step length, shortening width and improving cadence. Another finding is that lower staff to patient ratio and incorporating more than one patient at a time is both cost effective and time saving with optimum task focusing approach. Moreover practicing with other patients of similar impairments and difficulties was found to be motivating and helpful for patients however further studies should be conducted to investigate the effects of group training.

### Author`s Contribution

**QUA:** Conceived, designed and did statistical analysis & editing of manuscript.

**ANM:** Designed, did statistical analysis, review and final approval of manuscript.

**UH & SA:** Manuscript writing.

## References

[1] Eng JJ, Tang PF (2007). Gait training strategies to optimize walking ability in people with stroke: a synthesis of the evidence. Expert Rev Neurother.

[2] van de Port IG, Wevers L, Roelse H, van Kats L, Lindeman E, Kwakkel G (2009). Cost-effectiveness of a structured progressive task-oriented circuit class training programme to enhance walking competency after stroke: the protocol of the FIT-Stroke trial. BMC Neurol.

[3] MEMBERS WG, Go AS, Mozaffarian D, Roger VL, Benjamin EJ, Berry JD (2014). Heart disease and stroke statistics-2014 update: a report from the. Am Heart Assoc Circ.

[4] Pettersen R, Dahl T, Wyller TB (2002). Prediction of long-term functional outcome after stroke rehabilitation. Clin Rehabil.

[5] Veerbeek JM, Kwakkel G, van Wegen EE, Ket JC, Heymans MW (2011). Early prediction of outcome of activities of daily living after stroke. Stroke.

[6] McKevitt C, Fudge N, Redfern J, Sheldenkar A, Crichton S, Rudd AR (2011). Self-reported long-term needs after stroke. Stroke.

[7] van de Port IG, Kwakkel G, van Wijk I, Lindeman E (2006). Susceptibility to deterioration of mobility long-term after stroke. Stroke.

[8] Lord SE, McPherson K, McNaughton HK, Rochester L, Weatherall M (2004). Community ambulation after stroke: how important and obtainable is it and what measures appear predictive?. Arch Phys Med Rehabil.

[9] van de Port IG, Wood-Dauphinee S, Lindeman E, Kwakkel G (2007). Effects of exercise training programs on walking competency after stroke: a systematic review. Am J Phys Med Rehabil.

[10] Liesbet De Wit P, Putman K, Dejaeger E, Baert I, Berman P, Bogaerts K (2005). Use of Time by Stroke Patients.

[11] Wevers L, Van De Port I, Vermue M, Mead G, Kwakkel G (2009). Effects of task-oriented circuit class training on walking competency after stroke. Stroke.

[12] Dean CM, Richards CL, Malouin F (2000). Task-related circuit training improves performance of locomotor tasks in chronic stroke: a randomized, controlled pilot trial. Arch Phys Med Rehabil.

[13] Kim B, Park Y, Seo Y, Park S, Cho H, Moon H (2016). Effects of individualized versus group task-oriented circuit training on balance ability and gait endurance in chronic stroke inpatients. J Phys Ther Sci.

